# Disulfide-poly(β-amino ester) nanoparticles for glutathione-responsive drug delivery

**DOI:** 10.1039/d6ra07830c

**Published:** 2026-11-12

**Authors:** Camillo Colli, Sara Mullahi, Davide Moscatelli, Emanuele Mauri

**Affiliations:** a Department of Chemistry, Materials and Chemical Engineering “Giulio Natta”, Politecnico di Milano via E. Bassini 6 Milan 20133 Italy emanuele.mauri@polimi.it +39 0223993397

## Abstract

Stimuli-responsive polymeric nanocarriers have emerged as promising platforms for controlled drug delivery, enabling cargo release in response to specific biological signals. Among triggers, the high intracellular concentration of glutathione (GSH) offers a unique opportunity to activate redox-responsive systems through cleavage of disulfide bonds. In this work, a disulfide-containing poly(β-amino ester) (PBAE) was synthesized and conjugated with PEG to obtain block copolymers capable of spontaneous self-assembly into polymeric nanoparticles. While PBAEs have been previously established for nucleic acid delivery, their application as redox-responsive drug carriers for the physical encapsulation of small-molecule therapeutics remains largely unexplored. Here, we demonstrate that a disulfide-containing PEG–PBAE copolymer can efficiently encapsulate punicalagin (a model drug) through self-assembly, forming nanoparticles with hydrodynamic diameters below 200 nm and a near-neutral *ζ*-potential. These nanosystems showed pronounced redox-responsive behavior, with sustained drug release in the presence of GSH while limited release under physiological conditions (*ca.* 10%). The nanoparticles also exhibited good cytocompatibility at concentrations up to 0.2 mg mL^−1^. Overall, this study investigates the potential of combining the versatility of PBAEs with disulfide-mediated responsiveness to develop polymeric nanocarriers for controlled glutathione-triggered small-molecule drug release.

## Abstract 2

Self-assembled PEG–PBAE nanoparticles incorporating disulfide bonds enable glutathione-responsive degradation and on-demand release of physically encapsulated therapeutics.

## Graphical abstract

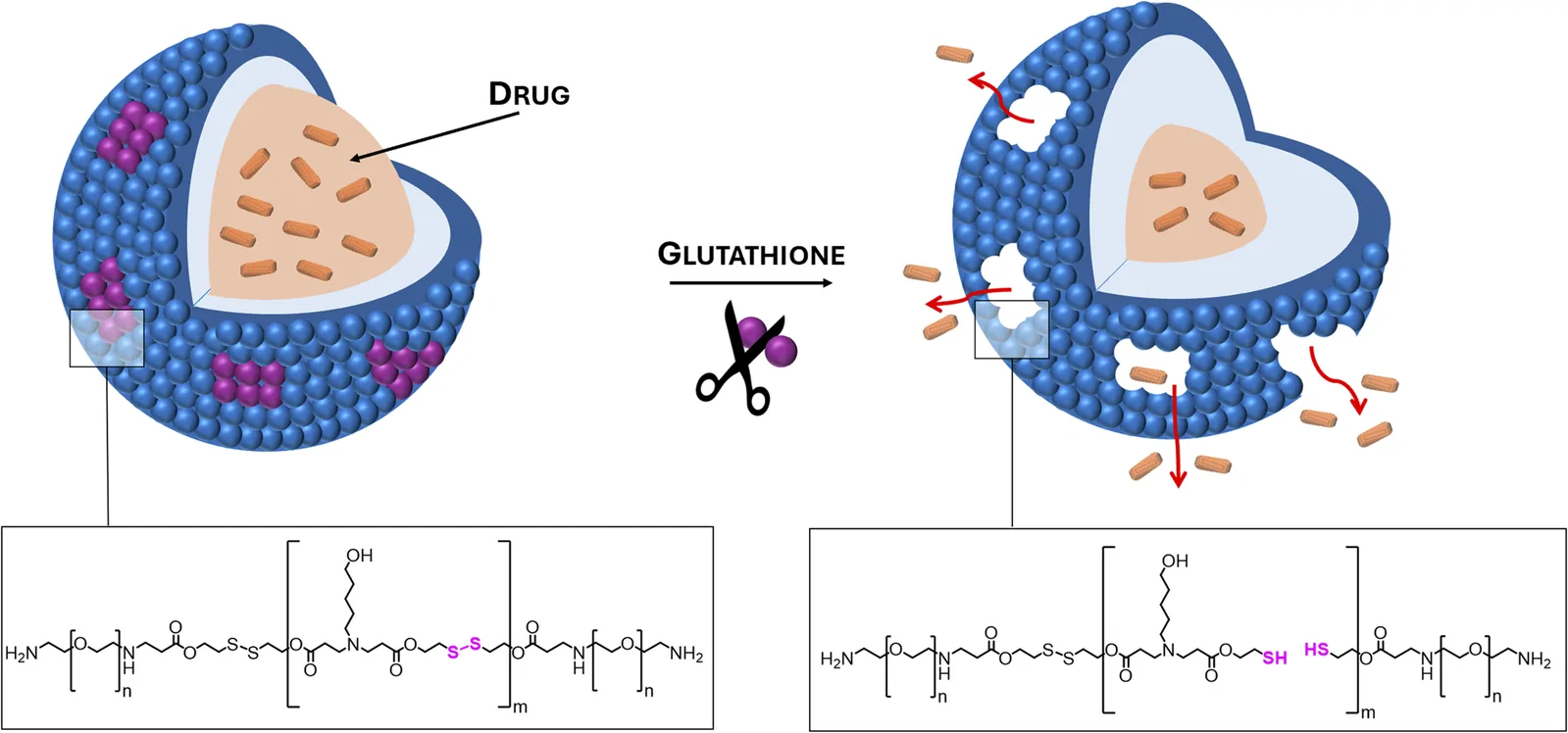

## 1. Introduction

In the framework of novel therapeutic treatments, design of nanocarriers for controlled drug delivery has emerged as a powerful tool for improving the efficacy of bioactive compounds, overcoming several limitations associated with conventional administration routes, such as poor bioavailability, rapid systemic clearance, and non-specific biodistribution.^1–4^ In particular, polymeric nanoparticles (NPs) have attracted considerable interest due to the wide range of synthetic approaches available for their formulation, which enable precise control over physicochemical properties such as size, surface characteristics, drug loading capacity, and degradation behavior.^5–11^ Among the different strategies explored in recent years, the design of stimuli-responsive NPs represents a leading approach to obtain smart delivery platforms capable of modulating their behavior in response to specific biological cues. These systems remain stable under physiological conditions while undergoing structural changes upon exposure to endogenous stimuli associated with cellular environments, such as pH variations, enzyme activity, oxidative stress, or altered redox conditions.^12–16^ Exploiting disease-specific biochemical signatures could lead to the selective activation of therapeutic systems and improve the treatment. A representative example is provided by the tumor microenvironment, where elevated intracellular concentrations of glutathione (GSH) arise as an adaptive response to persistent oxidative stress associated with rapid proliferation and metabolic dysregulation. In addition, GSH concentrations are typically in the low micromolar range in extracellular fluids and blood plasma, whereas intracellular cytosolic concentrations are generally in the millimolar range (approximately 1–10 mM).^17^ This concentration gradient provides a rationale for a selective cleavage of specific linkages following cellular internalization. For this reason, this distinctive biochemical feature has been exploited for the design of redox-responsive delivery systems.^18–23^ In particular, polymers containing disulfide bonds have attracted considerable interest because they remain relatively stable under physiological conditions while undergoing reductive cleavage upon exposure to intracellular GSH, thereby promoting payload release within cells. To date, many GSH-responsive delivery platforms reported to date rely on prodrug strategies, where therapeutic molecules are covalently linked to polymer or carrier components through disulfide-containing linkers.^24,25^ However, this approach requires chemical modification of the active molecule and depends on the restoration of its functional activity after bond cleavage, potentially restricting the range of therapeutics that can be used. An alternative strategy could be incorporating disulfide bonds directly into the nanocarrier structure: in this case, the therapeutic cargo can be physically entrapped within the NP and released following redox-induced carrier degradation, avoiding the need for drug derivatization and broadening the applicability of the delivery platform.^26–31^

Among biodegradable polymers employed for drug delivery, poly(β-amino ester)s (PBAEs) represent an attractive class owing to their structural versatility and tunable physicochemical properties. Their synthesis through Michael addition reactions enables straightforward modulation of polymer composition and degradation behavior, while the presence of protonable amine groups has promoted their extensive investigation as vectors for nucleic acid delivery.^32,33^ Beyond gene delivery applications, the physicochemical characteristics of PBAEs may also support the encapsulation and controlled release of small molecules as therapeutic compounds by exploiting electrostatic complexation, rather than relying on covalent prodrug strategies that require chemical modification of the active payload. In this context, the incorporation of disulfide-containing moieties within the PBAE backbone represents an appealing strategy to combine the advantageous formulation properties of PBAEs with redox-responsive behavior. This approach broadens the applicability of redox-responsive PBAEs while preserving the structural integrity and activity of the encapsulated drug and enables the development of smart nanocarriers that remain stable under physiological conditions while undergoing degradation in the reductive intracellular environment following cellular internalization. As a result, payload release can be selectively triggered by intracellular glutathione-rich environment, without requiring chemical modification of the therapeutic cargo.

In the present work, a disulfide-containing PBAE was synthesized and modified through PEG conjugation to design redox-responsive polymeric NPs.

This specific PEG modification was chosen referring to the work of Kim *et al.*,^34^ and intended to provide near-neutral *ζ*-potential through steric shielding, thereby enhancing the NP colloidal stability and the ability to cross biological barriers. Upon dispersion in aqueous media, the resulting polymer spontaneously self-assembled into nanosized particles with sizes below 200 nm, in the suitable range for biomedical applications.^35–38^

As a proof-of-concept therapeutic cargo, punicalagin, a polyphenolic compound from pomegranate, was selected because of its broad spectrum of therapeutic effects, including antioxidant, anti-inflammatory, antimicrobial, antiviral and antitumoral activities. This molecule presents an abundance of hydroxyl groups and aromatic rings, conferring an overall negative surface charge.^39–41^ Exploiting the interaction between the positively charged PBAE and the punicalagin molecular structure, which enables electrostatic and hydrogen-bonding interactions with cationic polymers, drug-loaded NPs were formed *via* self-assembly, avoiding the need for complex formulation procedures. The developed PEG–PBAE nanosystems were investigated in terms of physicochemical characteristics, glutathione-responsive behavior and drug release performance. A minimal drug leakage under physiological conditions (<10.5%) occurred, while up to 93% release was obtained in the presence of elevated GSH concentrations. Additionally, NP biocompatibility was assessed and confirmed at concentration below 0.2 mg mL^−1^. The findings highlight the potential of the synthesized disulfide-containing PEG–PBAE as a nanocarrier platform for the design of intracellular redox-responsive drug delivery systems.

## 2. Experimental section

### 2.1. Materials

5-Amino-1-pentanol (≥92%, MW = 103.16), acryloyl chloride (97%, MW = 90.51), ammonium hydroxide (30%, MW = 35.05), acetonitrile (ACN, ≥99.9%, MW = 41.05), dichloromethane (DCM, ≥99.8%, MW = 84.93), diethyl ether (≥99%, MW = 74.12), 4-(dimethylamino)pyridine (DMAP, ≥99%, MW = 122.17), dimethyl sulfoxide (DMSO, ≥99.9, MW = 78.13), ethyl acetate (≥99.5% MW = 88.11), l-glutathione reduced (GSH, ≥98%, MW = 307.32), hexane (≥99%, MW = 86.18), hydrochloric acid (HCl, 37%, MW = 36.46), methanol (MeOH, ≥99.9%, MW = 32.04), poly(ethylene glycol)_2000_ (PEG_2000_, average MW = 1900–2200), sodium sulfate (Na_2_SO_4_, ≥99%, MW = 142.04), thiazolyl blue tetrazolium bromide (MTT, 98%, MW = 414.32), were purchased from Merck KGaA (Darmstadt, Germany). 2-Hydroxyethyl disulfide (90%, MW = 154.24), formic acid (99%, MW = 46.03), *p*-toluenesulfonyl chloride (≥99%, MW = 190.64), sodium carbonate (Na_2_CO_3_, 98%, MW = 105.99), sodium hydroxide (≥97%, MW = 39.99), tetrahydrofuran (THF, 99.5%, MW = 72.11), triethylamine (TEA, 99%, MW = 101.19), were purchased from Thermo Fisher Scientific Inc. (Waltham, MA, USA). Chloroform-D_1_ + 0.03% TMS (CDCl_3_, ≥99.8%, MW = 120.38), dimethyl sulfoxide-d_6_ + 0.03% TMS (DMSO-d_6_, >99.8%, MW = 84.17), were purchased from FluoroChem Ltd (Hadfield, United Kingdom). Punicalagin (>98%, MW = 1084.72) was purchased from Biosynth (Staad, CH).

Milli-Q^®^ Direct system (Merck Darmstadt, Germany) was used to produce ultrapure water (DDIW) for the preparation of aqueous solutions. Solvents were of analytical grade and reagents were used as received.

### 2.2. 2-Hydroxyethyl disulfide diacrylate monomer synthesis (MSSA)

The synthesis of disulfide-containing diacrylate precursor (Fig. 1a) for redox-responsive NP fabrication was performed *via* acylation reaction, referring to the work of Hong *et al.*.^42^ Briefly, TEA (12.5 mL, 100 mmol) and 2-hydroxyethyl disulfide (6.2 g, 40.0 mmol) were dissolved in anhydrous THF (200 mL) and stirred for 15 min at 0 °C. Acryloyl chloride (18 g, 200 mmol) was then added dropwise to the mixture and the reaction was left under stirring for 20 h at room temperature (RT). The resulting triethylamine hydrochloride salts were removed by vacuum filtration and solvent evaporated under reduced pressure. The crude liquid product was dissolved in DCM (15 mL) and washed with an aqueous solution of Na_2_CO_3_ (0.1 M, 5 × 15 mL), then with DDIW (3 × 15 mL). The organic phase was dried over Na_2_SO_4_, filtrated under vacuum and the solvent removed under reduced pressure, obtaining a yellow liquid, which was stored in the dark at 4 °C until use. Chemical characterization was performed *via*^1^H-NMR in CDCl_3_. The resulting acrylate-terminated disulfide monomer is hereafter designated as MSSA (Monomer S–S Acrylate) throughout the manuscript.

**Fig. 1:**
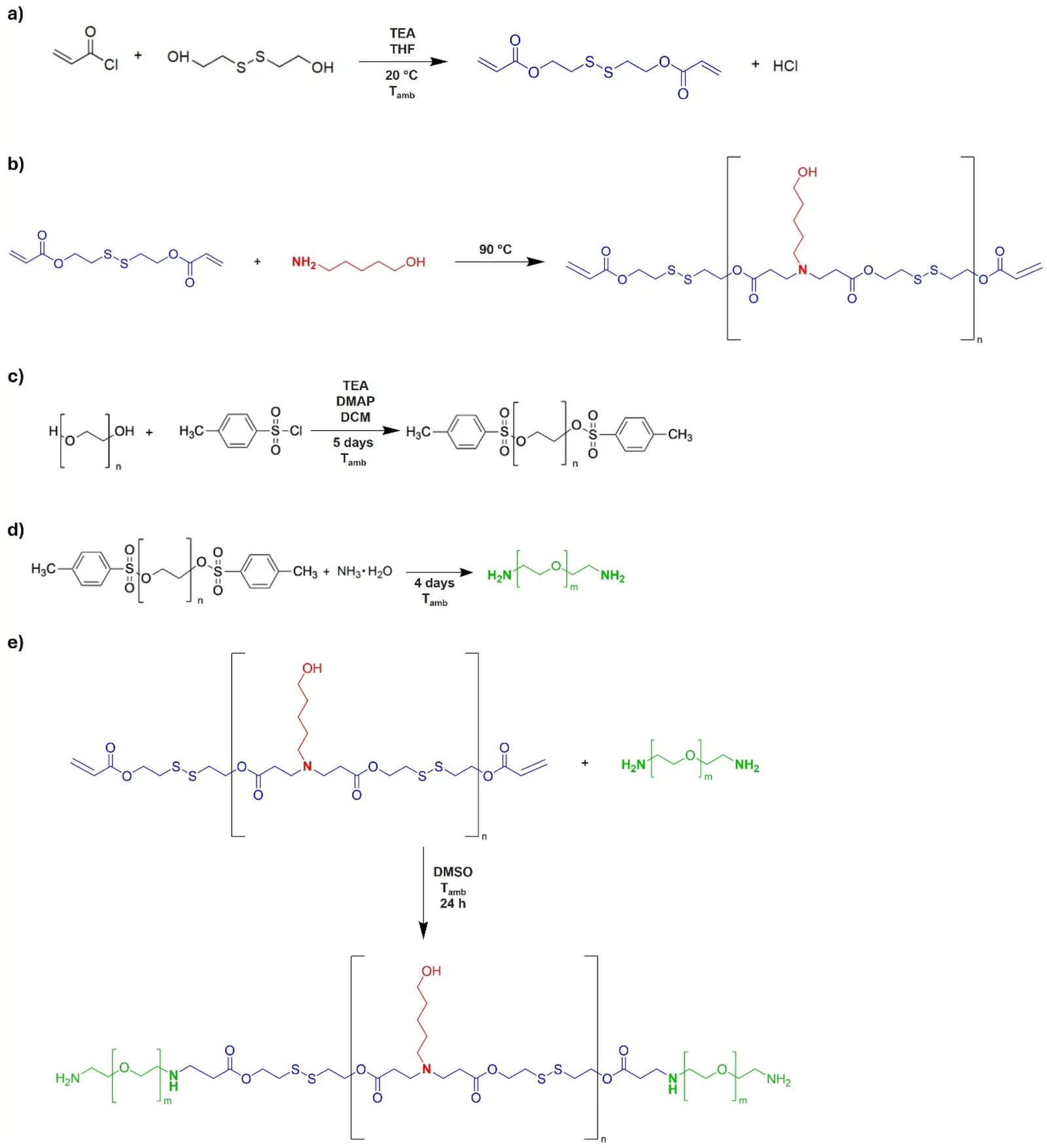
(a) MSSA acylation reaction synthesis from acryloyl chloride and 2-hydroxyethyl disulfide; (b) poly(β-amino ester) (PBAE) synthesis *via* Michael addition in a ratio 1 : 1 MSSA : 5-amino-1-pentanol; (c) PEG-Ts synthesis; (d) PEG-NH_2_ synthesis; (e) PEG-PBAE block-copolymer synthesis *via* aza-Michael addition.

### 2.3. Poly(β-amino ester)s synthesis (PBAEs)

PBAE was synthesized through a Michael-type addition reaction between the previously obtained MSSA diacrylate and 5-amino-1-pentanol. In this reaction, MSSA acted as the Michael acceptor, while 5-amino-1-pentanol served as nucleophile.^43–46^ The polymerization was performed under different reaction conditions to evaluate the effect of temperature and solvent volume on polymer yield and purity, with the aim of identifying the optimal synthesis parameters for a polymer (Fig. 1b) suitable for nanoparticle preparation. The experimental conditions for each reaction are summarized in Table 1. Briefly, MSSA (400 mg, 1.52 mmol) and 5-amino-1-pentanol (157.31 mg, 1.52 mmol), in a 1 : 1 stoichiometric ratio, were added to DMSO and left under stirring in the dark for at least 24 h. Then, the product was precipitated in diethyl ether (35 mL), followed by centrifugation to enhance the recovery, dried under nitrogen to remove all the residual solvent and stored at 4 °C until use. ATR-FTIR, ^1^H-NMR in DMSO-d_6_ and GPC analyses were performed to characterize the product.

**Table 1:** Experimental conditions for PBAE synthesis from MSSA and 5-amino-1-pentanol. Reactions were performed with a 1 : 1 MSSA : 5-amino-1-pentanol molar ratio

Sample labelling	*V*_DMSO_ [mL]	*T* [°C]	Reaction time [h]
PBAE-1	30	90	24
PBAE-2	1.5	90	24
PBAE-3	—	90	24
PBAE-4	30	90	96

### 2.4. Poly(ethylene glycol)_2000_ diamine synthesis (PEG-NH_2_)

Primary amine terminal groups were introduced on PEG_2000_ following the procedure proposed by Khatuntsev *et al.*,^47^ which involves the synthesis of PEG-tosylate derivative followed by ammonolysis (Fig. 1c and d).

#### 2.4.1. PEG-bis-tosylate synthesis

PEG_2000_ (3.83 g, 1.92 mmol), DMAP (23 mg, 0.19 mmol) and TEA (600 µL, 4.3 mmol) were dissolved in DCM (15 mL). In parallel, *p*-toluenesulfonyl chloride (2 g, 10.5 mmol) was dissolved in DCM (10 mL) and the resulting solution added dropwise to the PEG one, and left under stirring for 5 days at RT. Subsequently, the solvent was removed under reduced pressure, the residue was dissolved in ethyl acetate (15 mL) to precipitate triethylamine hydrochloride salts and filtered. The filtrate was recovered and the solvent was evaporated under vacuum. The product was redissolved in DCM (6 mL) and precipitated in diethyl ether (3 × 40 mL), to obtain PEG functionalized with terminal tosylate groups (PEG-Ts). Characterization was performed *via* ATR-FTIR and ^1^H-NMR in DMSO-d_6_.

#### 2.4.2. Ammonolysis

PEG-Ts (1 g, 0.43 mmol) was dissolved in ammonium hydroxide (10 mL) and left under stirring for 4 days at RT. The solvent was then removed under reduced pressure, and the resulting product was extracted with DCM (4 × 10 mL). The organic phases were combined, washed with brine (40 mL), dried over Na_2_SO_4_, filtered and concentrated under reduced pressure. The crude product was then resuspended in DCM (2 mL) and purified through precipitation in diethyl ether (25 mL). The resulting PEG-NH_2_ was recovered after solvent removal and characterized by ATR-FTIR and ^1^H-NMR in DMSO-d_6_.

### 2.5. PEG-PBAE block-copolymers synthesis

The reaction leading to the formation of PEG-PBAE block-copolymers was an aza-Michael addition^48,49^ between the acrylate-terminated PBAE chains and PEG-NH_2_. This reaction yielded an amphiphilic block copolymer consisting of a central PBAE segment and PEG chains at both termini (Fig. 1e), enabling the self-assembly in aqueous media and, consequently, the NP formation. PEG-NH_2_ (100 mg, 0.05 mmol) and PBAE were dissolved in DMSO (3.5 mL) in order to have a stoichiometric molar ratio PEG-NH_2_ : PBAE 2.5 : 1. The reaction was left under stirring for 24 h in the dark at RT. Then, the product was collected after precipitation in diethyl ether and purified through dialysis against DDIW (MWCO = 6–8 kDa). The final polymer was freeze-dried and characterized *via* ATR-FTIR and ^1^H-NMR in DMSO-d_6_; DLS/ELS analyses were performed after its resuspension in PBS.

### 2.6. Drug loading and release

A stock solution of punicalagin in DDIW (6 mg mL^−1^) was prepared and 100 µL of this solution added to freeze-dried PEG-PBAE block-copolymers (2 mg). After 10 min of incubation to promote polymer–drug association and NP self-assembly, 900 µL of DDIW were added, to obtain a nominal mass ratio of 0.3 mg_punicalagin_ mg_NP_^−1^. The resulting suspension was vortexed for 30 s and 100 µL placed in the internal chamber of a VivaSpin® (MWCO = 100 kDa, Sartorius AG, Göttingen, Germany) and dialyzed against 900 µL of exchange medium. Tests were performed under three different conditions: PBS, as a standard reference condition, low-GSH medium (10 µM GSH in PBS) representative of extracellular GSH concentration, and high-GSH medium (5 mM GSH in PBS), simulating the intracellular GSH levels.^17,50–52^ The encapsulation efficiency (EE%), was determined as follows: aliquots (3 × 100 µL) of the remaining suspension of drug–NPs were dried under gentle air flow, re-dispersed in 0.9 mL of DDIW, vortexed for 60 s and filtered through 0.22 µm nylon syringe filters; the supernatant was then collected and analyzed by HPLC (*λ* = 260 nm). EE% was calculated as reported in eqn (1):1
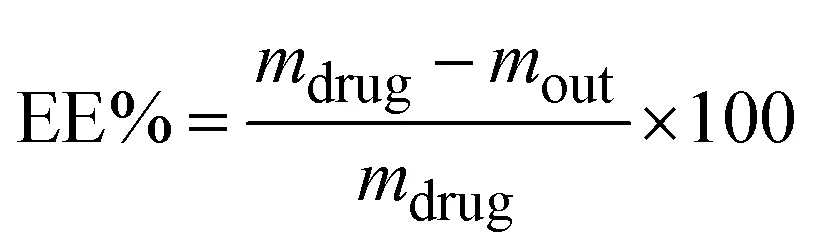
where *m*_drug_ is the total mass of added punicalagin and *m*_out_ is the non-encapsulated drug mass collected in the suspension. A calibration curve for punicalagin was defined *via* HPLC (*λ* = 260 nm), using drug solutions in DDIW ranging from 0.5 mg mL^−1^ to 100 ng mL^−1^ (SI, Fig. S1), and used as a reference for the estimation of non-encapsulated punicalagin.

The drug loading (DL%) was estimated according to eqn (2):2
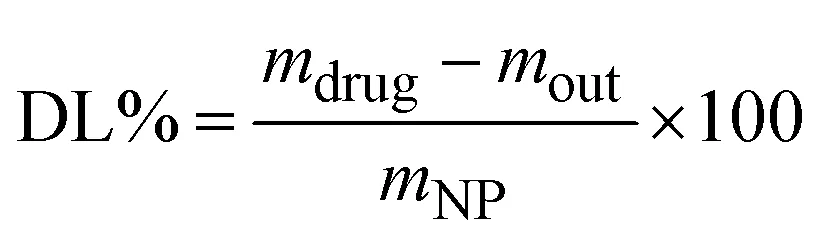
where *m*_NP_ is the mass of NPs.

The drug release profile was estimated at RT. To maintain sink conditions throughout the release study, drug-loaded NPs (4 µg_NP_ µL^−1^, prepared as mentioned above) were allowed to exchange against either PBS, low-GSH medium or high-GSH medium (900 µL). At defined timepoints, the entire release medium was collected for HPLC analysis (*λ* = 260 nm) and replaced with an equal volume of fresh medium, in order to avoid mass-transfer equilibrium with the surrounding solution. The cumulative percentage of drug released was calculated according to eqn (3):3
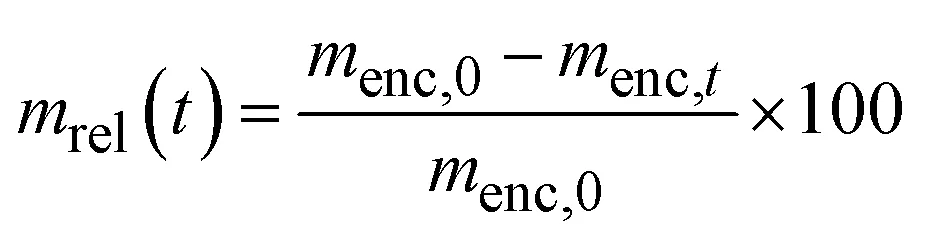
where *m*_rel_(*t*) is the percentage of drug released at time *t*, *m*_enc,0_ and *m*_enc,*t*_ are respectively the total amount of encapsulated punicalagin present in the nanoparticles formulation at the starting point (*t* = 0, according to DL%) and at time *t*. Experimental data were collected from three independent replicates and reported as mean ± standard deviation (SD).

### 2.7. Characterization techniques

#### 2.7.1 ^1^H-NMR analysis

Intermediate products and PEG-PBAE block-copolymers were characterized by proton nuclear magnetic resonance (^1^H-NMR) using a Bruker AC spectrometer (400 MHz, Bruker Corp., Billerica, USA). Samples were dissolved in CDCl_3_ or DMSO-d_6_ at concentration of 10 mg mL^−1^. Spectra were acquired using 64 scans. The chemical shifts were reported as *δ* values (ppm) with respect to trimethylsilylane (TMS) as the internal reference.

#### 2.7.2 ATR-FTIR analysis

Attenuated total reflectance Fourier transform infrared spectroscopy (ATR-FTIR) was performed using a Cary 630 spectrometer (Agilent Technologies Italia, Italy). Spectra were acquired at a resolution of 2 cm^−1^ in the wavenumber range of 4000–650 cm^−1^ at RT.

#### 2.7.3 GPC analysis

Molecular weight distributions of the PBAE polymer chains were analyzed using Size Exclusion Chromatography (SEC). The analyses were performed on an Agilent 1100 GPC/SEC system equipped with a PFG linear M column (PSS GmbH, Mainz, Germany), which was connected in series to an Agilent 1100 VWD/UV detector set at 300 nm, followed by a refractive index (RI) detector (Agilent Technologies). Samples were eluted with hexafluoroisopropanol (HFIP) at a flow rate of 1 mL min^−1^ and a column temperature of 40 °C. Calibration was conducted using polyethylene terephthalate (PET) standards (range 3500 Da–120 000 Da) to determine PET-equivalent molecular weight values.

#### 2.7.4 DLS/ELS analysis

The hydrodynamic diameter, polydispersity and *ζ*-potential of the synthetized NPs were determined by dynamic light scattering (DLS) and electrophoretic light scattering (ELS) using a Zetasizer Nano ZS (Malvern Instruments, Malvern, UK). Samples were dispersed in PBS at a concentration of 0.1 mg mL^−1^. Measurements were carried out at 25 °C and performed in triplicate, with results reported as mean values. To assess NP formation through self-assembly, the same DLS analyses were additionally conducted on samples dispersed in hexane under identical concentration and measurement conditions.

#### 2.7.5 HPLC analysis

Punicalagin quantification during NP-mediated release was performed by high performance liquid chromatography (HPLC) using an Agilent 1100 system (Agilent Technologies) equipped with a diode array detector set at *λ* = 260 nm. The separation was achieved on an Agilent Eclipse C18 column (250 mm length, 4.6 mm inner diameter) under gradient elution conditions. The mobile phase consisted of formic acid solution (1% in DDIW) and acetonitrile, according to the gradient program reported in Table S1 (SI). Analyzed samples consisted of the aqueous aliquots (900 µL) withdrawn at defined time points during the drug release experiments.

#### 2.7.6 TEM imaging

Transmission electron microscopy (TEM) imaging was exploited to analyze dry NPs dimensions after drug encapsulation, using a Philips CM200 Field Emission Gun (FEG). Micrographs were acquired at 200 kV. Samples were prepared by depositing a droplet of dispersion on a carbon-coated copper grid and air-dried at RT before observation. To obtain quantitative size distribution data, the diameters of 30 NPs across distinct micrographs were measured using Fiji ImageJ,^53^ and the resulting data were plotted as a size frequency histogram.

### 2.8. *In vitro* biocompatibility assessment

The biocompatibility of the developed NPs was evaluated through MTT assay using human dermal fibroblast (HDF, Merck KGaA, Darmstadt, Germany), in line with considerations outlined in ISO 10993-22.^54^ Cells were seeded in 96-well plates at a density of 6.5 ×10^3^ cells per cm^2^ and cultured in HDF medium, supplemented with 1 ng mL^−1^ fibroblast growth factor (recombinant human), 5 µg mL^−1^ insulin, 100 U mL^−1^ penicillin, and 0.1 mg mL^−1^ streptomycin. Empty NPs were suspended in cell medium at different concentrations (2 mg mL^−1^, 1 mg mL^−1^, 0.5 mg mL^−1^, 0.2 mg mL^−1^, 0.1 mg mL^−1^, 0.01 mg mL^−1^, 0.001 mg mL^−1^, 0.0001 mg mL^−1^) and incubated with cells for 24 h at 37 °C. Cell metabolic activity was assessed by exposure to MTT solution (0.5 mg mL^−1^ in PBS) for 3 h under standard culture conditions (37 °C, 5% CO_2_). The resulting absorbance was measured at 570 nm using a TECAN Spark plate reader (TECAN, Männedorf, Switzerland). To account for potential optical interference or non-specific light scattering caused by the polymeric chains at 570 nm, cell-free NP controls were included to assess background contributions to the absorbance signal. Untreated cells were used as reference control, and cell viability was expressed relative to the control group (*i.e.*, untreated cells). Experiments were performed in triplicate.

### 2.9. *In vitro* cell viability assessment in OVCAR3 cells following drug-loaded PEG–PBAE NPs administration

The potential therapeutic efficacy of punicalagin-loaded PEG–PBAE NPs were assessed using OVCAR3 cells, representative cell line of human ovarian carcinoma and characterized by high intracellular GSH levels.^55,56^ This cell line (ATCC® HTB-161™) was obtained from the American Type Culture Collection (LGC Standards, Teddington, UK) and cultured in RPMI 1640 medium (1×), supplemented with GlutaMax™ Supplement (Cat# 61870036; Thermo Fisher Scientific), 20% fetal bovine serum (Cat# F524-500 ML; Merck KGaA), and penicillin–streptomycin 100× solution (1%).

Cells were seeded in 96-well plates (6.5 × 10^3^ cells per well) and treated for 24 h with a concentration of free punicalagin of 200 µM,^57^ empty PEG–PBAE NPs (0.1 mg mL^−1^), and punicalagin-loaded PEG–PBAE NPs (0.1 mg mL^−1^). The cells were incubated at 37 °C under a humidified atmosphere with 5% CO_2_ and cell viability was determined *via* MTT assay as described in Section 2.8. Data were expressed as mean ± SD and experiments performed in triplicate.

### 2.10. Statistical analysis

Data were analyzed using Prism ver. 10.4.1 (GraphPad Software, San Diego, USA) and reported as mean ± SD unless otherwise specified. One-way analysis of variance (one-way ANOVA) followed by Tukey's multiple comparison test was used to assess statistical significance, with the threshold set at the level 0.05.

## 3. Results and discussion

### 3.1. MSSA characterization

The starting monomer for the synthesis of the redox-responsive PBAE polymer was prepared *via* a non-catalyzed acylation reaction between acryloyl chloride and 2-hydroxyethyl disulfide, yielding a bifunctional acrylate-terminated molecule. The resulting structure incorporates a disulfide bond, which was chosen to confer redox-responsive degradability to the final polymer network. Indeed, disulfide bridge is known to be readily cleaved in reductive environments, such as those characterized by elevated intracellular GSH concentration. The reaction afforded the desired product at 80% yield.

Structural confirmation was achieved through ATR-FTIR and ^1^H-NMR spectroscopy. The ^1^H-NMR spectrum (Fig. 2a) exhibited characteristic peaks consistent with the proposed structure: the alkene protons of the acrylate moiety were observed at 6.41 ppm (A), 6.14 ppm (B), and 5.87 ppm (A′); protons adjacent to the ester oxygen appeared at 4.43 ppm (C); and protons near the disulfide bond presented at 2.98 ppm (D). ATR-FTIR analysis (Fig. 3a) confirmed these findings, showing the alkene C=C stretching at 1650 cm^−1^ and bending at 809 cm^−1^, ester C=O stretching at 1737 cm^−1^, ester C–O stretching at 1172 cm^−1^, and C–S stretching near 700 cm^−1^. These characterizations support the successful synthesis of the diacrylate monomer with disulfide functionality, serving as a key building block for subsequent PEG-PBAE block-copolymer nanoparticle formation.

**Fig. 2:**
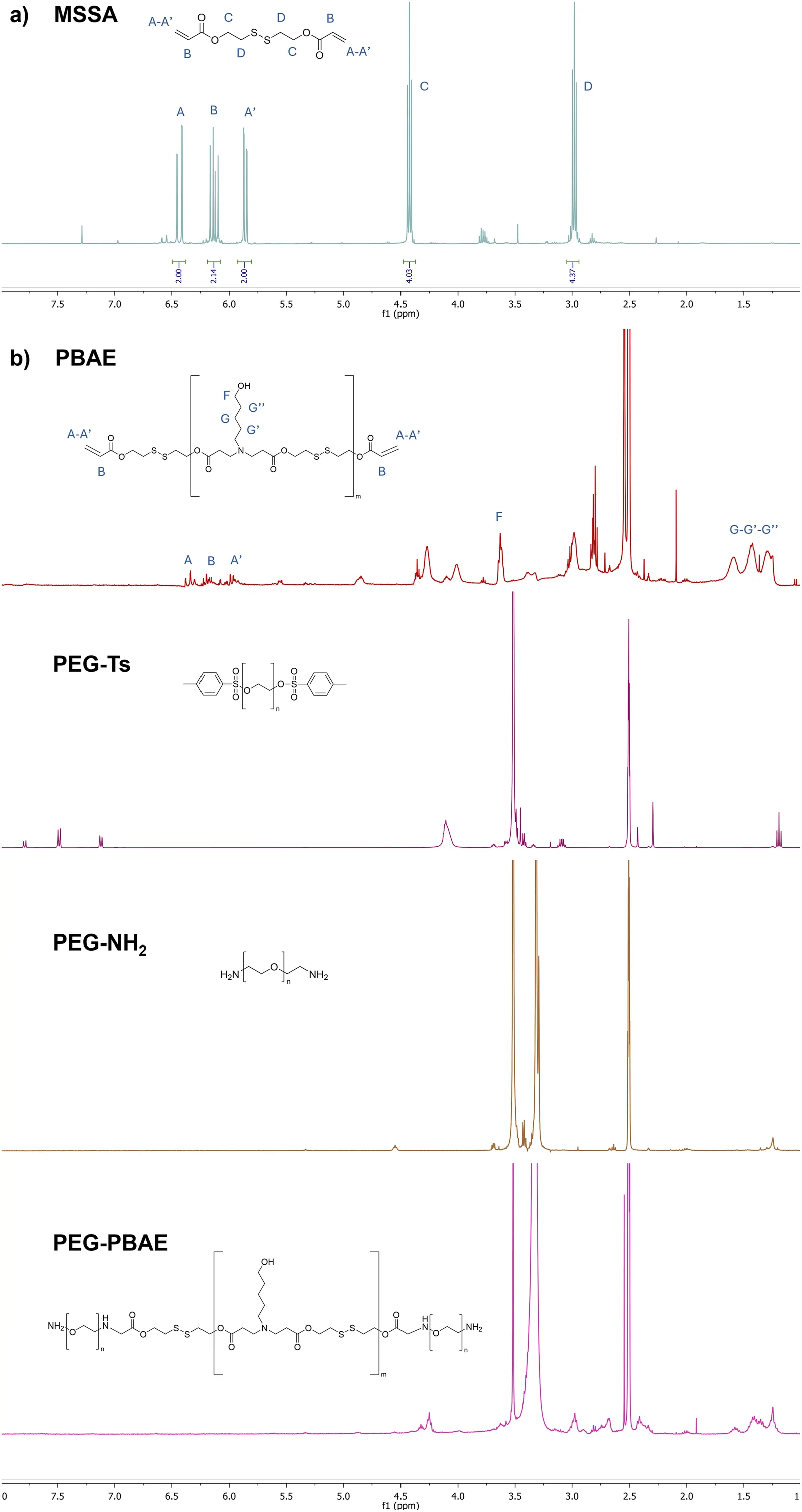
(a) ^1^H-NMR of MSSA in CDCl_3_ with the assigned peaks in blue. (b) ^1^H-NMR of PBAE-3 in DMSO-d_6_ with the assigned peaks in blue (red line), PEG-Ts (purple line), PEG-NH_2_ (brown line), PEG-PBAE block-copolymer (pink line) with the acrylate peaks missing (red box highlighting the region between 5.5 and 7.5 ppm), validating complete PEG end-capping.

**Fig. 3:**
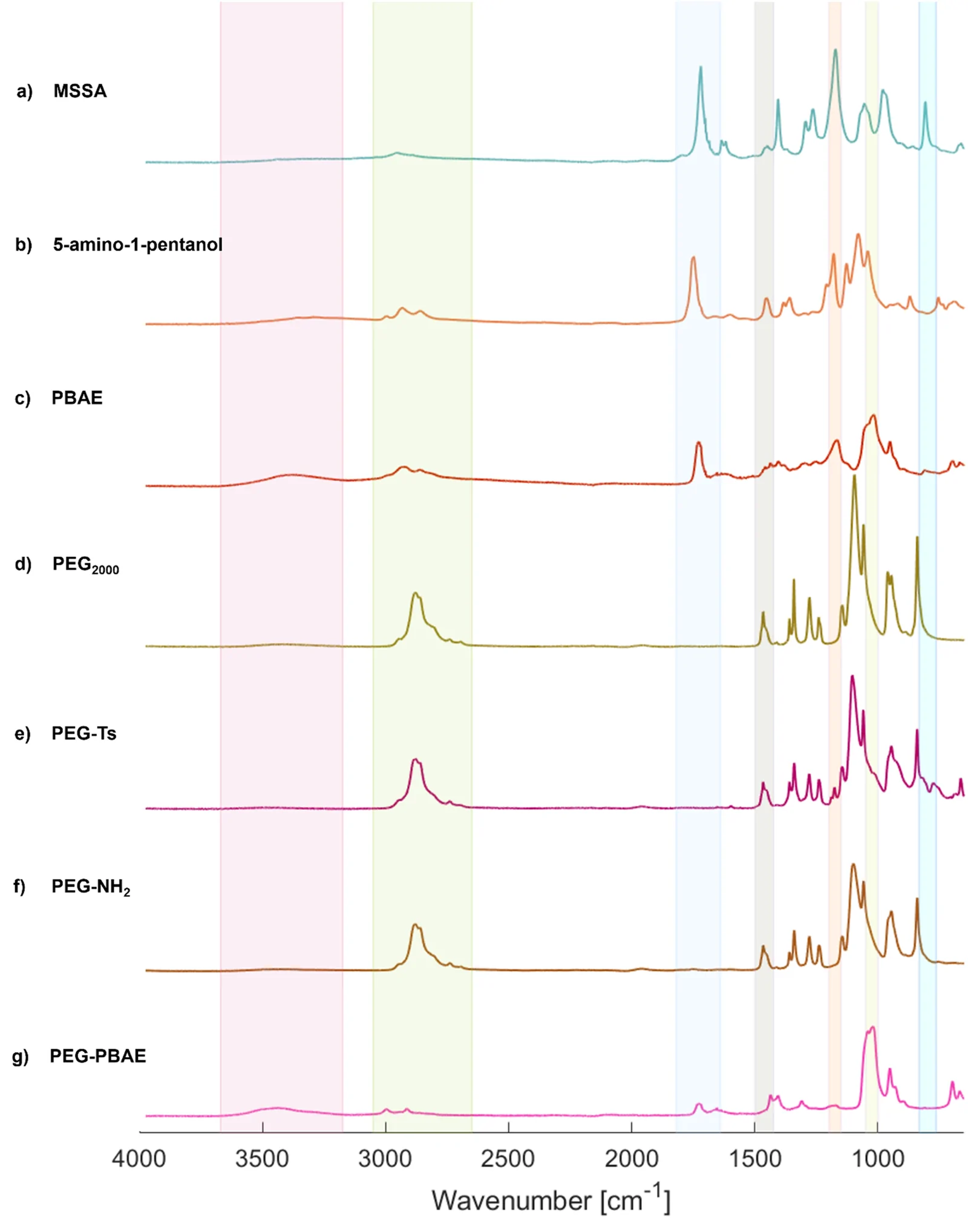
ATR-FTIR spectra comparison in PEG-PBAE synthesis intermediates with the characteristic peaks highlighted. From top to bottom: (a) MSSA (cyan line), (b) 5-amino-1-pentanol (orange line), (c) PBAE-3 (red line), (d) PEG_2000_ (green line), (e) PEG-Ts (purple line), (f) PEG-NH_2_ (brown line), (g) PEG-PBAE (pink line). The different functional group bands interested in the modification of the compounds are highlighted.

### 3.2. PBAE synthesis and characterization

The synthesized MSSA monomer reacted with 5-amino-1-pentanol, *via* Michael-type addition to form the PBAE polymer. The polymerization was optimized by varying two parameters: reaction time and solvent content, progressively reducing the latter down to solvent-free conditions, in which the liquid MSSA served as the reaction medium. These parameters were selected based on their direct influence on the extent of polymerization and molecular weight distribution in step-growth systems.^58–60^ Specifically, longer reaction times and reduced solvent volumes could both increase monomer-to-chain-end encounter frequencies between MSSA and 5-amino-1-pentanol reactive groups. This promotes the sequential bis-addition onto the diacrylate monomer, leading to the formation of higher-molecular-weight PBAEs with narrower molecular weight distribution. Conditions yielding low PDI were retained for subsequent NP formulation, as a dispersity near the theoretical step-growth limit indicates a more controlled chain length distribution, which is expected to favor low NP polydispersity during formulation.^61,62^

The reaction temperature was kept constant at 90 °C across all experiments and was therefore not considered as an additional variable. This choice is supported by literature evidence indicating that, in catalyst-free thermal aza-Michael step-growth polymerizations, reducing the temperature significantly slows the reaction kinetics, resulting in lower monomer conversions at equivalent reaction times. Critically, the bis-addition step, which governs effective chain extension and is inherently slower than the initial mono-addition, is disproportionately penalized at lower temperatures.^63–65^ Maintaining 90 °C ensured that both addition steps proceeded efficiently, maximizing the probability of reaching the high conversion regime required to obtain PDI values approaching 2.

In addition, the resulting PBAEs were designed to incorporate tertiary amine groups, which confer a cationic character to the polymer and underpin its ability to engage electrostatic interactions with negatively charged molecules.^66,67^ The protonation of these amines at physiological-relevant pH has been extensively explored in formulating NPs for gene delivery, where electrostatic complexation with negatively charged nucleic acids is functional for an efficient encapsulation of the payload.^39,68^ Here, we extend this structural feature to the physical entrapment of small-molecule therapeutics for their delivery, where the polymer cation character was expected to enhance encapsulation efficiency through electrostatic interactions with the payload, complemented by hydrogen bonding and hydrophobic contributions.

The molecular weight of each synthesized PBAE was evaluated through GPC (SI, Fig. S2), and the obtained results in terms of molecular weight averages and PDI are summarized in Table 2. Notably, polymers PBAE-1 and PBAE-2 exhibited broader molecular weight distributions and higher polydispersity. This suggests a heterogeneous mixture of polymer chain lengths, which could negatively impact on the uniformity, size distribution and self-assembly behavior of the resulting polymeric NPs. These differences among PBAEs could be related to the synthesis conditions and the extent of monomer conversion achieved (Table 1). Since the aza-Michael addition proceeds *via* a bimolecular, concentration-dependent step-growth mechanism, the bulk reaction (*i.e.*, PBAE-3) and the extended-duration reaction (*i.e.*, PBAE-4) could reach a higher degree of conversion than the diluted, 24 h reactions (*i.e.*, synthesis of PBAE-1 and PBAE-2). At lower conversion, unreacted oligomers coexisted with more extended chains, leading to higher apparent molecular weight distribution of the resulting polymer.

**Table 2:** Weight-average molecular weights (*M*_w_) and polydispersity index (PDI) results of PBAEs synthesized *via* Michael addition. Analysis was conducted with GPC/SEC using hexafluoro-isopropanol on PFG linear M column

Sample	*M*_w_ [Da]	PDI [—]
PBAE-1	1.45 × 10^4^	10.8
PBAE-2	3.41 × 10^4^	12.2
PBAE-3	2.69 × 10^5^	4.2
PBAE-4	3.87 × 10^5^	1.8

Moreover, the presence of solvent during synthesis appears to influence molecular weight variability and conversion, consistent with a monomer dilution effect and differential chain mobility in solution. This reaction parameter lowers encounter frequencies and different diffusion/reaction pathways within the 24 h reaction window, thus yielding polymers of diverse molecular weights. In contrast, PBAE-3 and PBAE-4 display both higher molecular weights and narrower molecular weight distributions compared to the previous samples, reflecting the distinct roles of solvent removal and extended reaction time in driving chain growth and modulating molecular weight distribution.

Specifically, PBAE-4 was synthesized over a longer reaction period (4 days) at 90 °C and showed a molecular weight and PDI indicative of well-advanced chain growth and a more homogeneous length distribution, consistent with progressive consumption of available reactive end-groups (acrylates and amines) beyond what is achievable within 24 h of reaction. Hence, the extended reaction time emerges as a relevant parameter of dispersity control, allowing the polymerization to approach a more uniform growth regime. PBAE-3, synthesized under solvent-free conditions within 24 h, achieved comparably high molecular weights through increased monomer-to-chain-end encounter frequency;^69,70^ the absence of solvent, while highly effective in promoting chain growth, results in a molecular weight distribution that, although broader than the step-growth theoretical limit, represents a substantial improvement over solvent-based syntheses conducted at shorter reaction times. Overall, PBAE-3 and PBAE-4 polymers demonstrated the most favorable combination of molecular weight and dispersity among the synthesized polymers, and were therefore selected as candidates for NP formation.

^1^H-NMR analysis confirmed the successful synthesis of the disulfide-containing PBAEs. A representative spectrum of PBAE-3 is reported in Fig. 2b and revealed the key structural signals of the synthesized polymers. In particular, the peaks corresponding to the acrylate protons can be detected at 6.35 ppm, 6.20 ppm and 5.98 ppm (A–B–A′), reflecting the vinyl end motifs of the polymer chain. Signals attributable to methylene groups adjacent to ester oxygen atoms (4.35 ppm) and sulfur atoms (2.7–2.9 ppm) are consistent with the disulfide-containing structure, and additional peaks relative to the amino-alcohol segment derived from 5-amino-1-pentanol are observed in the regions expected for methylene groups neighboring nitrogen, hydroxyl, and aliphatic moieties.^71,72^ Moreover, the ^1^H-NMR spectrum is consistent with the expected polymer architecture and the formation of tertiary amines, as indicated by the signals of methylene protons adjacent to the tertiary nitrogen in the 2.4–2.8 ppm range, which partially overlap with those of the methylene protons next to the disulfide bond.^73^

ATR-FTIR analysis further confirmed polymer formation, providing complementary structural evidence: Fig. 3c shows spectra of PBAE-3 compared with 5-amino-1-pentanol (Fig. 3b) and MSSA (Fig. 3a). The spectrum of MSSA shown the characteristic acrylate peaks, including the C=C alkene stretching around 1650 cm^−1^ and bending at 809 cm^−1^.^72,74^ Additionally, the ester carbonyl stretching was observed at 1737 cm^−1^, the C–O ester stretching at 1172 cm^−1^ and the C–S bond near 700 cm^−1^ confirming the presence of disulfide linkage. The spectrum of 5-amino-1-pentanol showed the expected broad O–H/N–H stretching region between 3200 and 3400 cm^−1^ together with characteristic C–O stretching at 1082 cm^−1^. Following polymerization, the PBAE spectrum retained the absorption bands associated with ester, hydroxyl, amine, and disulfide-containing moieties, confirming preservation of the functional groups. At the same time, a reduction in the intensity of the acrylate-related bands (at both 1650 and 809 cm^−1^) was observed, consistent with the consumption of vinyl groups during the aza-Michael addition reaction. These spectral changes collectively confirm the successful synthesis of the disulfide-containing PBAE while preserving the disulfide group content.

### 3.3. PEG-NH_2_ characterization

PEG was introduced to the terminal ends of the PBAE chains to exploit its hydrophilic character and steric stabilization properties, promoting the formation of NPs with physicochemical properties suitable for biomedical applications.^75–77^

PEG with a molecular weight of 2000 Da was selected based on literature findings,^34,78,79^ which highlighted that the conjugation to cationic polymers (including PBAE) can modify their amphiphilic character, improving aqueous dispersibility, stability, biocompatiblity and promoting controlled self-assembly.^49^ In particular, the PEG molecular weight affects these properties and the NP formation/performance. Low molecular weight PEG (*e.g.*, 0.8–2 kDa) enabling a dense layer formation leads to reduced non-specific interactions and improves NP penetration. Conversely, high molecular weight PEG generates excessive steric hindrance that can impair cellular uptake and endosomal escape. However, PEG effectiveness jointly depends on both chains' length and their spatial arrangement, as well as their conjugation point, and a trade-off between these parameters is essential to simultaneously achieve high mobility, colloidal stability, and reduced aggregation and opsonization.^80,81^

Amine-terminated PEG, required for the subsequent aza-Michael conjugation reaction, was synthesized through a two-step functionalization process involving tosylation followed by nucleophilic substitution with ammonia.

ATR-FTIR and ^1^H-NMR analyses confirmed successful polymer modification.

In the ^1^H-NMR spectra of PEG-Ts, and amine-terminated PEG (Fig. 2b), the PEG backbone's methylene protons appear at 3.5 ppm (–C*H*_2_C*H*_2_O–). The PEG-Ts spectrum shows peaks at 2.43 ppm corresponding to the tosyl methyl groups and the doublets between 7.0–8.0 ppm from the aromatic ring, confirming tosyl group incorporation. Successful ammonolysis is confirmed by the complete disappearance of tosyl-associated signals in the PEG-NH_2_ spectrum.

ATR-FTIR spectra (Fig. 3d–f) further confirmed the polymer step-by-step modifications. The disappearance of the broad O–H stretching at 3425 cm^−1^ observed in pristine PEG indicates successful end-group substitution. Sulfonyl stretching bands at 1177 cm^−1^ and 1030 cm^−1^ appear in PEG-Ts, validating the first functionalization step, and were no longer distinguishable in PEG-NH_2_. In the latter, weak bands in the N–H stretching (3300–3500 cm^−1^) and N–H bending (approximately 1550 cm^−1^) regions were compatible with the presence of primary amine termini.

### 3.4. PEG-PBAE synthesis and NP formation

The polymer constituting the NPs was synthesized *via* an aza-Michael addition between the amino termini of PEG-NH_2_ and the vinyl termini of the PBAE polymer, yielding the PEG-PBAE conjugate. This polymer features a central PBAE block characterized by a cationic character arising from the protonatable tertiary amines introduced by 5-amino-1-pentanol units during polymer synthesis, and functional for electrostatic complexation of negatively charged cargo. The PEG chains at the polymer termini, instead, sterically shield the cationic part upon NP self-assembly. The amphiphilic character of the copolymer is expected to drive its self-assembly NP after dispersion in aqueous media, and in presence of negatively charged cargo, electrostatic interactions with the protonatable amines of the PBAE core are expected to contribute to NP formation and stabilization, with controlled size and surface charge. Thus, PEG functionalization was supportive of NP stability while maintaining efficient loading of negatively charged therapeutic agents. This synthesis strategy effectively combines the functional benefits of PBAE's cationic core with PEG's steric stabilization for optimizing drug delivery applications.^82,83^

As mentioned in Section 3.2, PBAE-3 and PBAE-4 were selected for PEG conjugation and the subsequent NP synthesis due to their favorable characteristics determined *via* GPC (*i.e.*, lower PDI and a monomodal molecular weight distribution with a high average molecular weight). The resulting PEG-PBAE block-copolymer was chemically characterized to confirm the effective synthesis. Fig. 2b shows the ^1^H-NMR spectra of PBAE-3 (as representative of the used PBAE samples, green line), PEG-NH_2_ (orange line), and the resulting PEG-PBAE-3 block-copolymer (labelled as PEG-PBAE, brown line). The characteristic peak of PEG backbone at 3.57 ppm is detectable in the block-copolymer spectrum, confirming successful PEG incorporation. Signals attributable to the PBAE backbone, including methylene protons adjacent to ester groups (4.4–4.2 ppm) and protons α to the disulfide bond (2.7–2.9 ppm), are also preserved in the PEG-PBAE block-copolymer spectrum. Notably, the alkene proton signals between 5.9 and 6.5 ppm, visible in the PBAE spectrum, are absent in the copolymer spectrum, indicating successful aza-Michael addition at the vinyl termini. Additionally, integration of the PEG (methylene protons) and PBAE (methylene protons adjacent to the tertiary nitrogen) signals in the purified block-copolymer was consistent with the incorporation of PEG blocks at both chain ends of the PBAE, in line with the expected architecture.^84^

In Fig. 3, the ATR-FTIR spectra of PBAE-3 (red line), PEG-NH_2_ (brown line), and PEG-PBAE (pink line) are shown. Both PBAE and PEG-PBAE exhibit O–H stretching band, respectively at 3384 cm^−1^ and 3431 cm^−1^; aliphatic C–H stretching appears in the 2723–3046 cm^−1^ range, while the ester carbonyl (C=O) and C–O stretching bands are observed at 1727 cm^−1^ and 1172 cm^−1^, respectively. The tertiary amine C–N stretching band at 1312 cm^−1^ is present in both polymers. Moreover, the PEG-PBAE spectrum features an additional weak N–H stretching band at 3273 cm^−1^, attributable to the conjugated PEG amine groups, and the disappearance of the acrylate C=C bands, confirms the conjugation between PBAE and PEG-NH_2_.

Upon dispersion in PBS, the structural rearrangement of the copolymer lead to the formation of the NPs, characterized though DLS and ELS to evaluate the hydrodynamic diameter and *ζ*-potential. These measurements were performed on NPs obtained from the two PEG-PBAE block-copolymers synthesized using PBAE-3 and PBAE-4, respectively, and summarized in Table 3. NPs derived from PBAE-3 (*i.e.*, NP1) showed a monodisperse size distribution with a hydrodynamic diameter of 154.4 nm and a low PDI (0.11). The *ζ*-potential of +0.78 mV (*i.e.*, near-neutral value) reflects the steric shielding provided by the PEG, with a possible shift the hydrodynamic shear plane outward, reducing the measured surface potential while the underlying cationic character of the PBAE backbone is retained, preserving the electrostatic cargo complexation capacity of the PBAE core.^85,86^ Similarly, NP2, synthesized from PBAE-4, displayed similar monodisperse size (122.1 nm), low PDI and *ζ*-potential characteristics. The slightly lower hydrodynamic diameter compared to NP1 might be attributed to the monodispersity of PBAE-4, since the molecular weight of the two polymers is comparable, yielding a smaller hydrodynamic size and lower PDI (SI, Fig. S3). Notably, despite the different molecular weight distribution of PBAEs, both formulations produced NPs with comparably narrow size distributions, suggesting that the process effectively normalizes polymer polydispersity during particle formation.

**Table 3:** Dynamic light scattering (DLS) and electrophoretic light scattering (ELS) results of three different NPs analyzed. Starting materials were used as the best candidates from PBAE-3 and PBAE-4

Sample	Composition	*D*_H_ [nm]	PDI [—]	*ζ*-potential [mV]
NP1	PBAE-3, PEG-NH_2_	154.4	0.11	0.78
NP2	PBAE-4, PEG-NH_2_	122.1	0.05	0.44

The self-assembly process was further confirmed by dispersing PEG-PBAE block-copolymers in a nonpolar aprotic solvent (*i.e.*, hexane) where no nanoparticle formation was detected, as evidenced by DLS measurements revealing random Brownian motion of free polymer chains (SI, Fig. S4). This highlights the critical role of the polar aqueous environment and electrostatic interactions for driving nanoparticle assembly.^87,88^ Based on these findings, PEG-PBAE NP2, due to their smaller size, were selected for subsequent drug release and biocompatibility studies.

### 3.5. Drug loading and release

The redox-responsive performance of the synthetized PEG-PBAE NPs was evaluated using punicalagin as a model drug to assess the encapsulation efficiency and triggered release behavior. HPLC analysis revealed an encapsulation efficiency (EE%) of 95.8%, while the drug loading (DL%) was 14.4%. DLS and ELS analyses showed a slight increase in both the average hydrodynamic diameter (147.5 nm) and the *ζ*-potential (3.1 mV) following punicalagin encapsulation. TEM confirmed the range size and a round shape aspect of the NPs (Fig. 4a), with an average diameter of 115 ± 16 nm in the dried state (Fig. S6). This value is in agreement with the DLS hydrodynamic diameter measured in PBS, with the slightly smaller size observed by TEM likely attributable to dehydration and collapse of the hydrophilic PEG shell upon drying under vacuum.

**Fig. 4:**
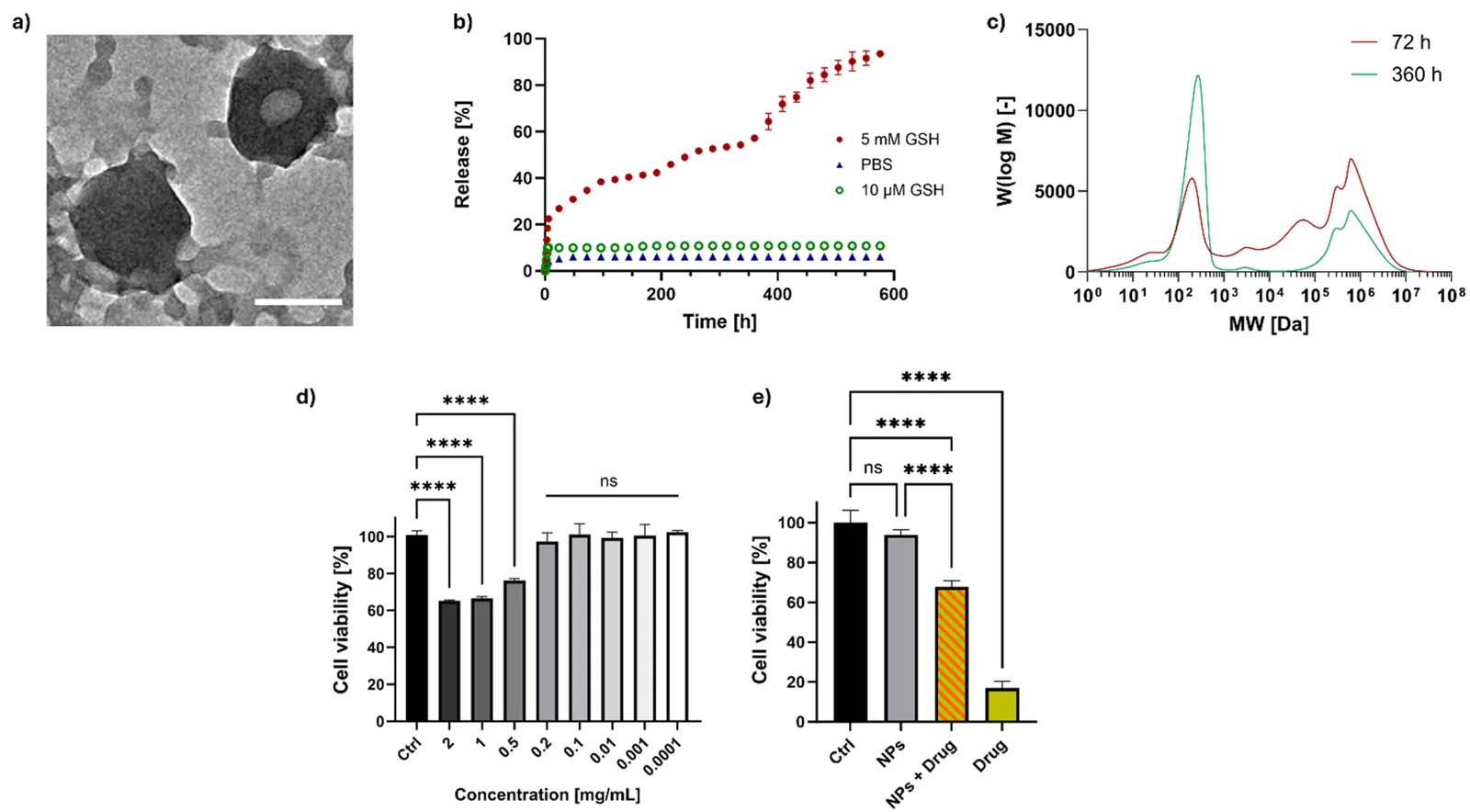
(a) TEM micrograph of PEG-PBAE NP2 after drug encapsulation; scale bar = 100 nm, acquisition at 200 kV. (b) Punicalagin release profiles from NP2. The three analyzed conditions show different drug release profiles between PBS/extracellular GSH concentration (10 µM GSH medium) and GSH intracellular concentration (5 mM GSH medium). (c) GPC analysis of PEG-PBAE block-copolymer in 5 mM GSH solution over time: analysis was performed at two different timepoints (72 h and 360 h), presenting a gradual degradation of the redox-responsive polymer. (d and e) *In vitro* cytotoxicity MTT assay of: (d) empty PEG-PBAE NP2 tested on HDF cell line; (e) empty PEG-PBAE NP2 (NPs, *C*_NPs_ = 0.1 mg mL^−1^), punicalagin-loaded PEG-PBAE NP2 (NPs + drug, *C*_Drug_ = 200 µM, *C*_NPs_ = 0.1 mg mL^−1^) and free punicalagin (drug, *C*_Drug_ = 200 µM), tested on OVCAR3 cells, representative of cell line with relevant intracellular GSH level. In all groups, the cytotoxic effects are expressed in terms of cell viability levels normalized against internal controls (*i.e.*, untreated cells, in black, Ctrl) measured through the MTT assay. The results are reported as the mean ± SD of three independent experiments. Statistical analysis was performed using one-way ANOVA. *p < 0.05, **p < 0.01, ***p < 0.001, ****p < 0.0001.

To investigate the GSH-responsive release behavior of punicalagin-loaded PEG–PBAE NPs and distinguish GSH-associated release from baseline release in the absence of a reducing agent, release profiles were evaluated in three different media: PBS (pH 7.4), as a reference condition, low-GSH medium (PBS containing 10 µM GSH), representing an extracellular physiological GSH level,^17,52^ and high-GSH medium (PBS containing 5 mM GSH), simulating the intracellular/tumor GSH concentration.^50^

Release profiles exhibited significant differences between the three media (Fig. 4b). In PBS and low-GSH medium (10 µM), punicalagin release was low and reached a plateau value respectively at approximately 6.5% within 48 h and 10.4% within 24 h, indicative of limited passive diffusion through the polymer network and the slight degradability of disulfide linkage under neutral/physiological conditions. Conversely, in the presence of high-GSH medium, release was markedly accelerated with an initial burst followed by a sustained release overtime, reaching an almost complete release after 24 days (approximately 94%). In particular, *ca.* 35% of the loaded drug was released within the first 48 h, while release under PBS remained negligible over the same timeframe. The release profile was consistent with the redox-responsive nature of the NPs, suggesting that the accelerated drug release observed under GSH conditions mimicking the intracellular environment can be associated with NP disulfide bond cleavage.

The sustained rather than instantaneous release profile observed under high-GSH medium can be explained by considering the presence of interactions between the partially protonated tertiary amines of the PBAE backbone and the phenolic moieties of punicalagin, including electrostatic interactions, hydrophobic/aromatic-aliphatic interactions, and hydrogen bonding, which likely contribute to retaining the drug during polymer degradation and modulating its subsequent release. The initial release phase can be attributed to the cleavage of disulfide bonds in more accessible regions of the NP structure, promoting the release of readily accessible drug molecules. In contrast, the subsequent, sustained release phase may reflect progressive disassembly of the PBAE backbone, associated with further cleavage of multiple disulfide bridges distributed throughout the polymer structure, leading to matrix destabilization and the gradual release of the remaining encapsulated drug.

Indeed, upon exposure to high GSH concentration, cleavage of the disulfide bonds generates lower-molecular-weight thiol-terminated polymer fragments, facilitating nanoparticle disassembly and drug release.^33,34,83^ The occurrence of this degradation process was supported by both HPLC and GPC analyses. HPLC data revealed the appearance of degradation products following incubation in GSH medium (Fig. S8) and GPC analysis performed after 72 and 360 h showed new peaks at lower molecular weights indicative of the fragmentation in shorter polymeric chains (Fig. 4c). This confirms the redox responsiveness of the material and its selective degradation in reducing solutions.

### 3.6. NP cytotoxicity

The cytotoxicity of the synthesized PEG-PBAE NPs was evaluated by MTT assay using human dermal fibroblasts (HDFs). This cell line was selected as a model cells to assess the cellular response to the polymeric carrier. The results are summarized in Fig. 4d. At higher NP concentrations (from 2 to 0.5 mg mL^−1^), a significant reduction in cell viability was observed, with decreasing values up to 38% relative to untreated controls. In contrast, no statistically significant differences in cell viability were observed at concentrations of 0.2 mg mL^−1^ and below, indicating that the empty PEG–PBAE NPs were well tolerated by HDFs at these concentrations.

Furthermore, the polymer degradation analyses (Section 3.5 and Fig. 4c) showed that exposure to GSH promoted the formation of lower-molecular-weight polymer fragments, consistent with the reductive cleavage of disulfide bonds within the PBAE backbone. A similar process could occur in the presence of intracellular GSH. Considering the cellular response observed for the NPs at concentrations of 0.2 mg mL^−1^ and below, it is reasonable to hypothesize that the resulting polymer fragments may not cause substantial additional cytotoxic effects under these conditions. This hypothesis is consistent with previous studies on redox-responsive disulfide-containing nanocarriers, which reported cellular tolerance of low-molecular-weight thiol-containing degradation products, after GSH-mediated cleavage of S–S linkages.^50^ Overall, these findings demonstrate that the PEG-PBAE NPs exhibit acceptable biocompatibility at concentrations up to 0.2 mg mL^−1^, which fall within concentration ranges commonly explored *in vitro* for polymeric nanocarriers,^89,90^ supporting their suitability for further biological investigations. Referring to these results, we performed an *in vitro* preliminary investigation of the potential therapeutic outcome of the punicalagin-loaded NPs on tumor cells OVCAR3 (Section 3.7).

### 3.7. Cell viability assessment in OVCAR3 cells treated with punicalagin-loaded PEG–PBAE NPs

The cellular response to punicalagin-loaded NPs was evaluated in OVCAR3 *via* MTT assay (Fig. 4e). Empty PEG–PBAE NPs, tested at 0.1 mg mL^−1^, maintained cell viability at 94%, with no statistically significant difference compared with untreated controls, indicating no relevant cytotoxic effects of the carrier at the concentration tested. Treatment with drug-loaded nanosystems reduced cell viability to 67.5% (****p < 0.0001 *vs.* Ctrl and ****p < 0.0001 *vs.* NPs), and free punicalagin resulted in a more pronounced reduction in cell viability, to 19% (****p < 0.0001 *vs.* Ctrl).

These results indicate that the PEG–PBAE nanocarriers were tolerated at the tested concentration, while the punicalagin-loaded formulation retained a measurable effect on OVCAR3 cells. Considering that the same nominal punicalagin concentration was used in both the free and encapsulated forms, the stronger effect observed with free drug could be related to its immediate availability in the culture medium, whereas encapsulation limited the amount of drug immediately available to cells. Furthermore, the response to the loaded NPs, together with the enhanced drug release previously observed under high-GSH condition, was consistent with the proposed redox-responsive behavior of the formulation. Given the elevated GSH levels typically found in the intracellular environment,^55,91^ these findings provide an indication that GSH-mediated release contributed to the biological response observed with the drug-loaded NPs. Although further studies are required to directly verify the mechanism, we can hypothesize that intracellular GSH acts as a trigger for drug release through the cleavage of disulfide bonds in the polymer backbone, contributing to the observed response.

## 4. Conclusions

This study investigates the feasibility of synthesizing disulfide-containing PEG–PBAE copolymers and their use for the formation of self-assembled, redox-responsive NPs. The incorporation of S–S linkages within the polymer backbone enables glutathione-triggered responsiveness, exploiting a naturally occurring intracellular reducing agent to induce structural destabilization of the nanocarrier.

The synthetized PEG–PBAE systems efficiently encapsulated the model compound punicalagin and formed NPs with hydrodynamic diameters below 200 nm, consistent with dimensions typically considered suitable for biomedical drug delivery systems. Drug release studies showed a redox-dependent behavior, consistent with GSH-triggered polymer degradation, with significantly enhanced release in the presence of GSH (*ca.* 94% drug release) compared to neutral buffer and physiological conditions, where release remained limited.

*In vitro* cytocompatibility studies on human dermal fibroblasts showed that the developed NPs were well tolerated at concentrations up to 0.2 mg mL^−1^. Furthermore, the preliminary evaluation in OVCAR3 cells showed that the punicalagin-loaded NPs retained biological activity, providing a proof-of-concept for their application in drug delivery. The observed cellular response was consistent with a GSH-mediated drug release in the intracellular environment. Overall, these findings support the potential of disulfide-containing PEG–PBAE NPs as a redox-responsive platform for drug delivery.

## Author contributions

CC: methodology, data curation, formal analysis, investigation, validation, writing – original draft; SM: investigation, data curation; DM: resources, visualization; EM: conceptualization, methodology, supervision, visualization, resources, writing – review and editing.

## Conflicts of interest

There are no conflicts of interest to declare.

## Supplementary Material

RA-OLF-D6RA07830C-s001

## Data Availability

The data supporting this article have been included as part of the supplementary information (SI). Additional data are available upon request from the authors. Supplementary information is available. See DOI: https://doi.org/10.1039/d6ra07830c.
