# Supplementary material for: Disulfide-poly(β-amino ester) nanoparticles for glutathione-responsive drug delivery
Source: RSC Adv. 2026 Nov 12. Online ahead of print. doi: 10.1039/d6ra07830c (PMC13756900; doi:10.1039/d6ra07830c)
Supplement: Supplementary file 1 [file RA-OLF-D6RA07830C-s001.pdf]

Supplementary Information (SI) for RSC Advances.
This journal is © The Royal Society of Chemistry

SUPPLEMENTARY INFORMATION

Disulfide-poly(β-amino ester) nanoparticles for glutathione-responsive drug delivery

*Camillo Colli [a], Sara Mullahi [a], Davide Moscatelli [a], Emanuele Mauri [a]**

[a] Department of Chemistry, Materials and Chemical Engineering "Giulio Natta", Politecnico di Milano, via E. Bassini 6, 20133 Milan, Italy

*corresponding author Tel.: +39 0223993397; e-mail: emanuele.mauri@polimi.it

Punicalagin HPLC calibration curve

Figure S1 shows the calibration curve obtained *via* HPLC analysis of samples ranging from 0.5 mg/mL to 100 ng/mL (7 points). Table S1 presents the gradient program utilized for punicalagin HPLC analysis, using a C18 column (250 mm length, 4.6 mm inner diameter) and ACN + Formic acid (1% v/v in DDIW)

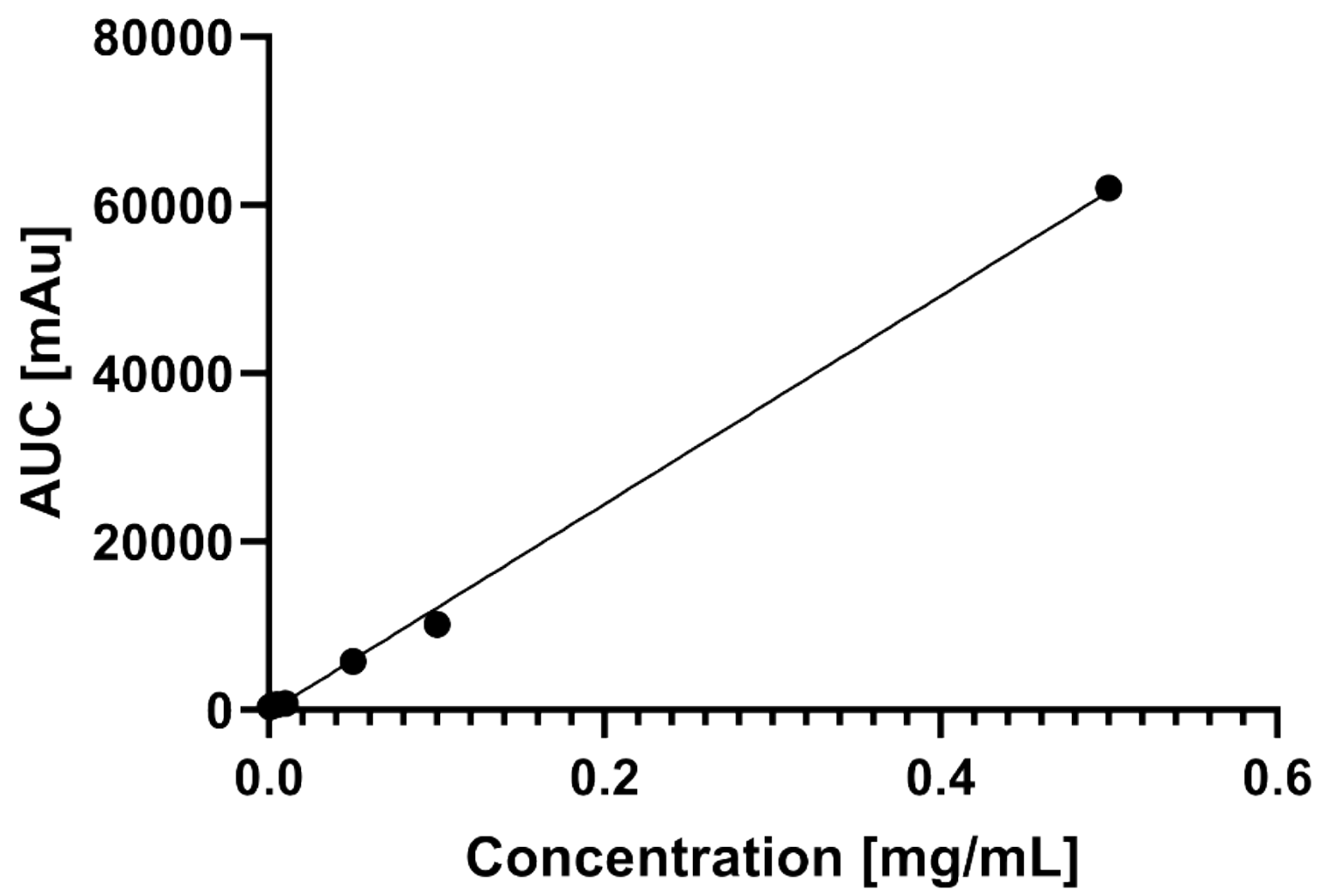

as mobile phases with a flowrate set at 1 mL/min.

Figure S1. HPLC calibration curve for punicalagin. Linear fit: m = 123067, R^2 = 0.9986.

Table S1. HPLC method for punicalagin analysis. Gradient analysis has been performed with acetonitrile (ACN and formic acid 1% solution in DDIW as mobile phases). Flowrate fixed at 1 mL/min.

Time [min]	Formic acid 1% [%]	ACN [%]
0	95	5
18	85	15
20	35	65
25	95	5
30	95	5

GPC analysis of PBAEs

Figure S2 presents the molecular weight distribution plots obtained from GPC analysis of synthesized PBAEs.

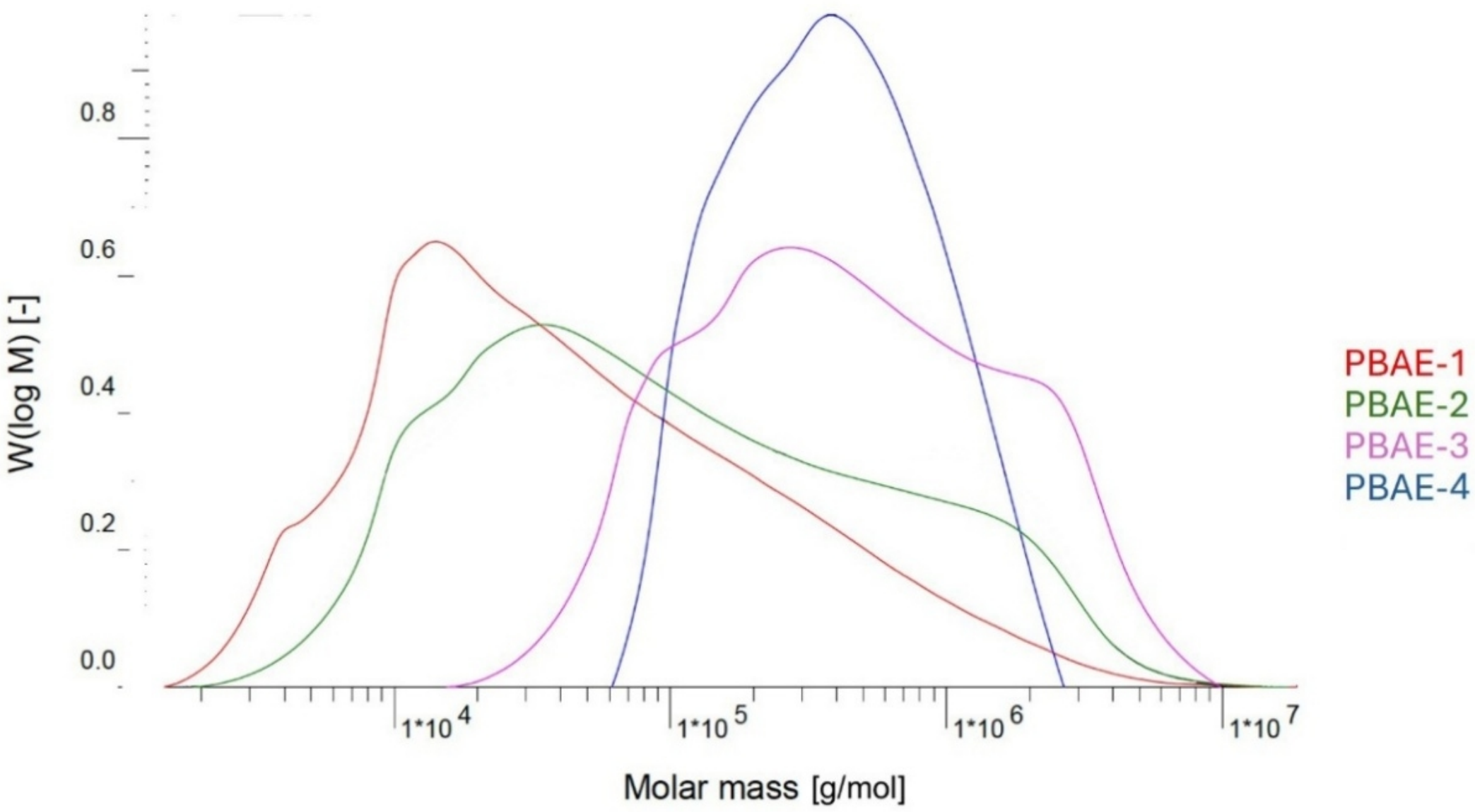

Figure S2. Raw GPC/SEC chromatogram of synthesized PBAEs. On the right the legend of the curves is presented.

Molecular weight and dispersity values were obtained by conventional SEC calibration against linear PET standards, should therefore be regarded as relative PET-equivalent values rather than absolute molar masses. As discussed in the literature, the relationship between hydrodynamic volume and molar mass may vary between the calibration standards and the analyzed polymers, particularly when their architectures deviate from those of the linear calibrants [1–3]. This effect may be relevant for the PBAEs investigated here, which feature lateral amino-alcohol chains that may influence their hydrodynamic behavior. Such

variations may affect the apparent molar mass distribution and contribute to an overestimation of the calculated dispersity.

DLS measurements of NP1 and NP2

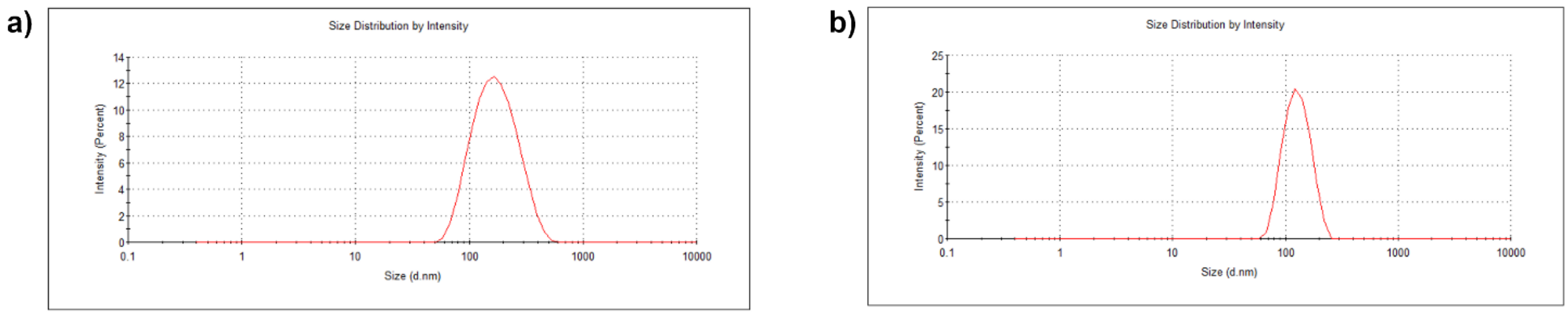

Figure S3. DLS analysis of (a) NP1 and (b) NP2, showing a narrow polydispersity and monodisperse population of self-assembled NPs.

Figure S3 presents the raw data of DLS analysis of self-assembled PEG-PBAE NPs, dispersed in PBS. The two figures show (a) NP1 and (b) NP2 analysis, confirming the monodisperse population of self-assembled NPs, with low PDI.

PEG-PBAE self-assembly confirmation via DLS analysis

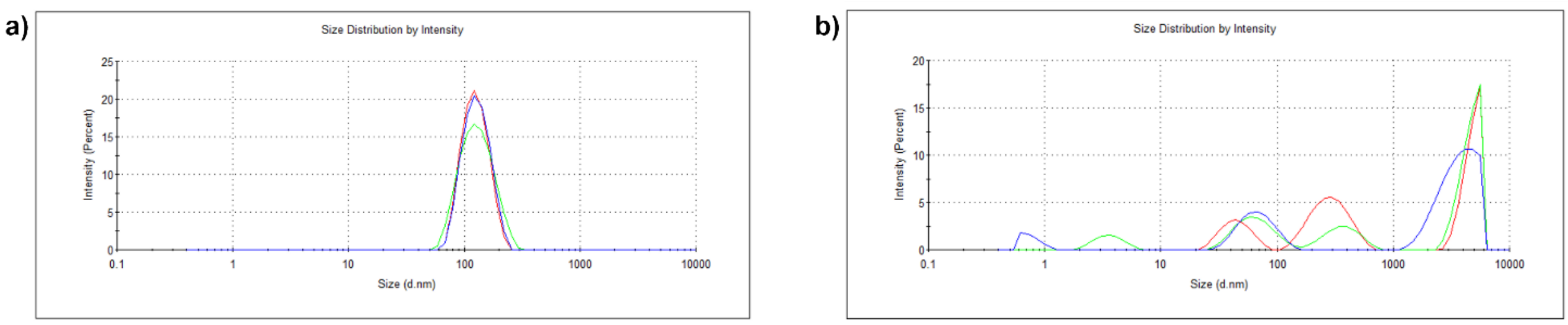

Figure S4. DLS measurements of PEG-PBAE in (a) PBS (polar protic solvent), (b) n-hexane (apolar aprotic solvent). Analysis performed in n-hexane presents random chain movement during three different runs of the same samples, confirming the capability of PEG-PBAE self-assembly in polar protic solvents only.

To verify the self-assembly of the PEG–PBAE copolymers in PBS (Figure S4a), DLS measurements were also performed after dispersing the polymer in the aprotic apolar solvent *n*-hexane (Figure S4b). Unlike the well-defined monodisperse NP population observed in PBS with a low PDI, measurements in *n*-hexane showed highly variability and random chain movement, with a lack of reproducible trends among consecutive acquisitions, indicating the absence of stable NP formation. These findings support that self-assembly of the PEG–PBAE copolymers occurred in aqueous media, according to the nature of the polymer.

Other DLS analysis of PEG-PBAE NPs

Figure S5 presents the analyses performed in PBS on other PEG-PBAE NPs, based on high polydispersed PBAEs synthesized during this study. The behavior of formed NPs presents a bimodal or a polydispersed distribution of the NPs, due to the high polydispersity of the starting PBAE.

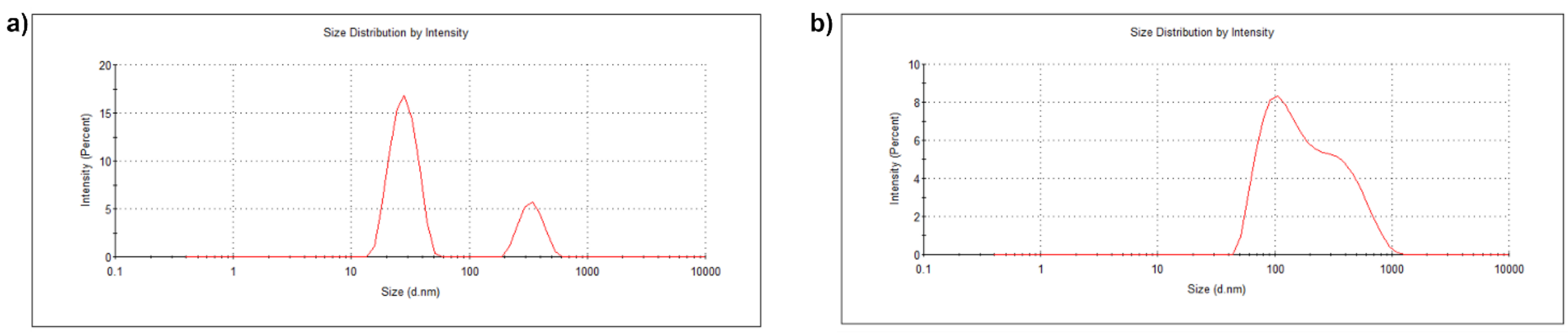

Figure S5. DLS measurements of high-polydispersed PEG-PBAE in PBS. (a) PEG-PBAE deriving from PBAE-1 produced a bimodal distribution of NPs; (b) PAG-PBAE deriving from PBAE-2 produced a polydispersed distribution of NPs.

TEM micrographs analysis

Representative TEM micrograph of punicalagin-loaded PEG–PBAE NP2 were acquired and analyzed using ImageJ software to obtain a NP size histogram. Figure S6 shows the corresponding particle size distribution histogram calculated from 30 individual NPs on different micrographs, confirming DLS results.

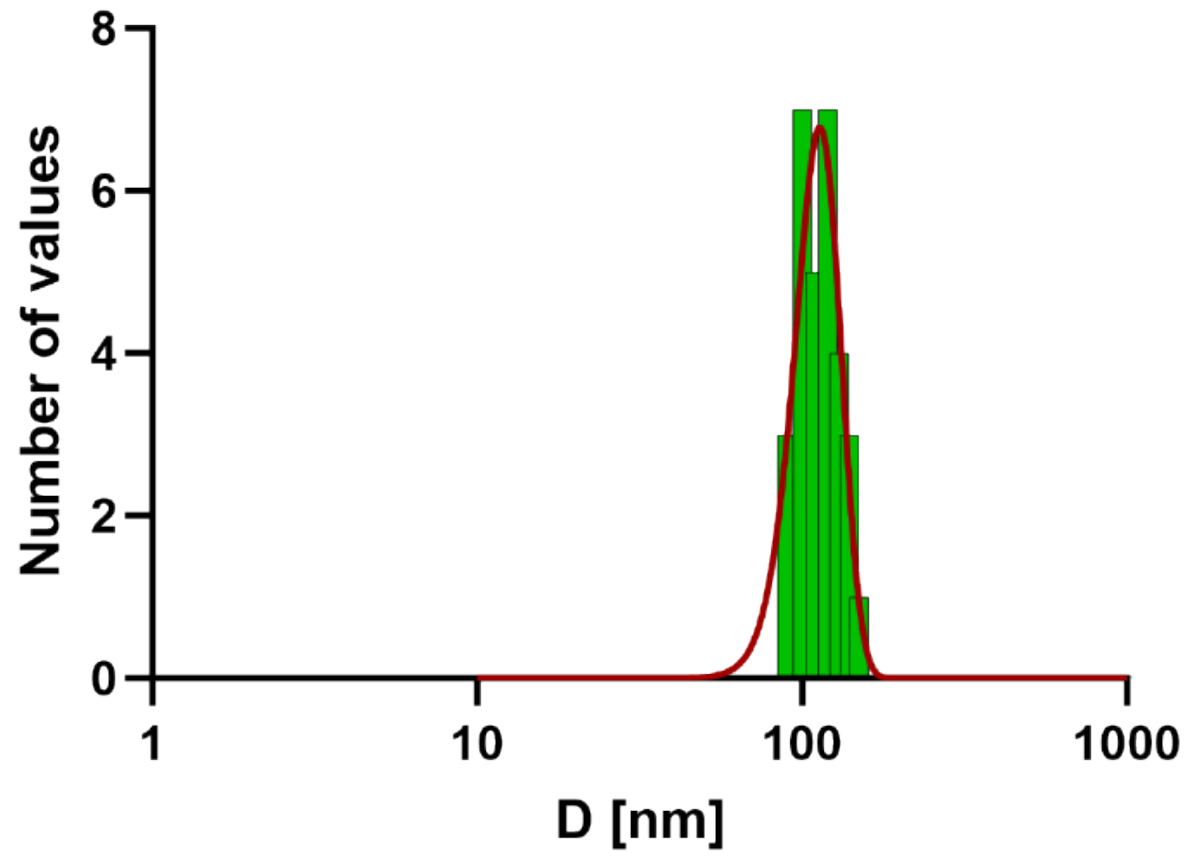

Figure S6. Histogram distribution analysis of TEM NPs micrographs. D = 115 ± 16 nm.

Control experiment for drug release study

To assess whether the dialysis membrane could limit the drug release kinetics measured for the NPs, a control experiment was performed using free (non-encapsulated) punicalagin under the same release conditions. As shown in Figure S7, more than 97% of the free punicalagin crossed the membrane within 24 h. This result indicates that the membrane did not represent a major barrier to punicalagin diffusion under the experimental conditions and supports the interpretation that the slower release observed from the NPs was primarily associated with the polymer architecture and the GSH effect.

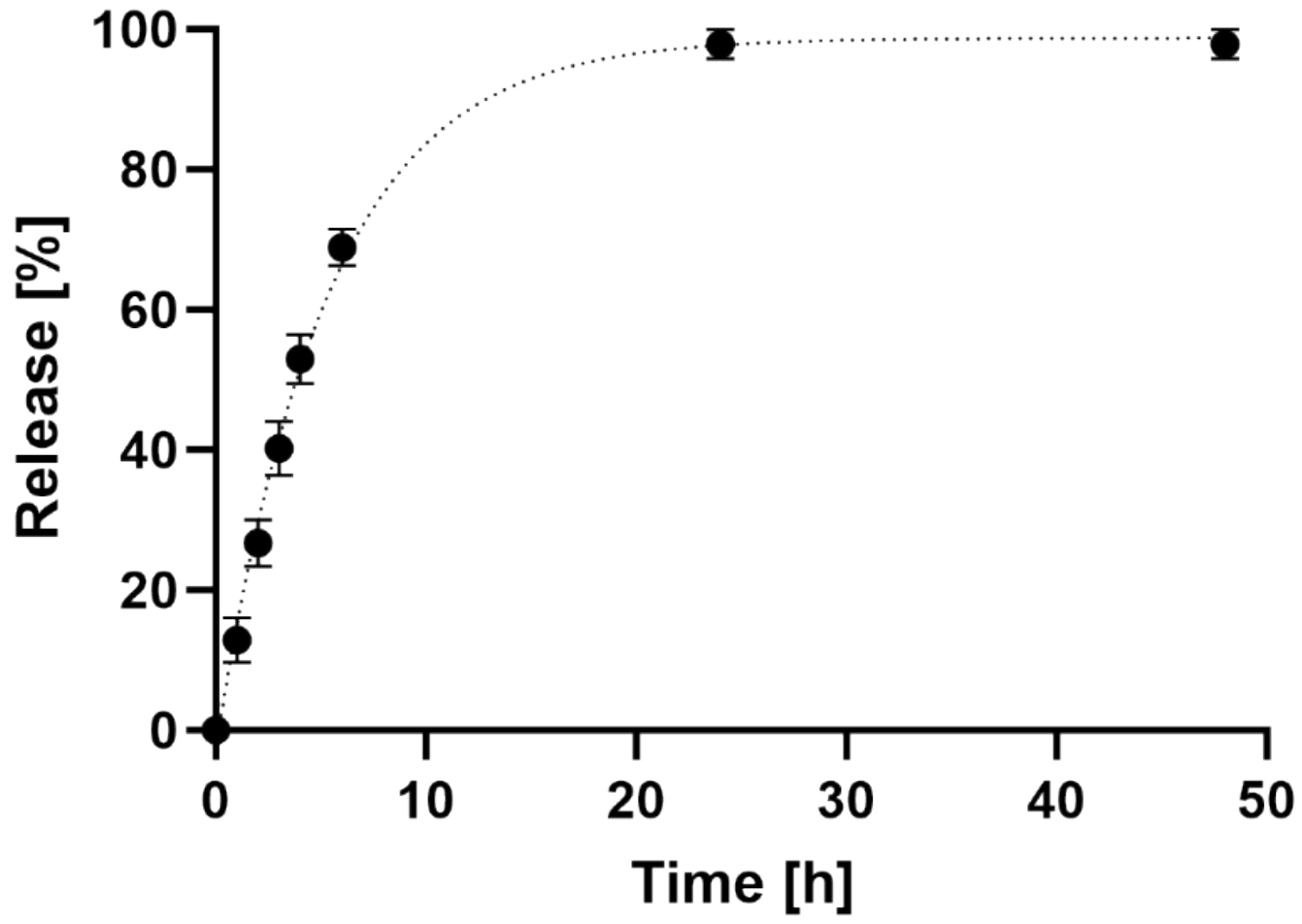

Figure S7. Release test of free punicalagin solution across Vivaspin membrane (MWCO = 100 kDa), showing a complete release (>97%) in less than 24 h.

HPLC peak analysis

Figure S8 shows the raw HPLC spectra of punicalagin drug release tests analyzed in calibration curve, drug release (at 48 h) and the end of drug release tests. The two distinct peaks of punicalagin isomers (α, β) are present in the elution time range 9-13 min, while in the initial time of the spectra (3-4 min) new peaks are

appearing, referring to the low-molecular weight products generated from the disulfide bond cleavage of the polymeric backbone. Since these peaks are well separated from the punicalagin peaks, no interference in the signal integration for drug release is present.

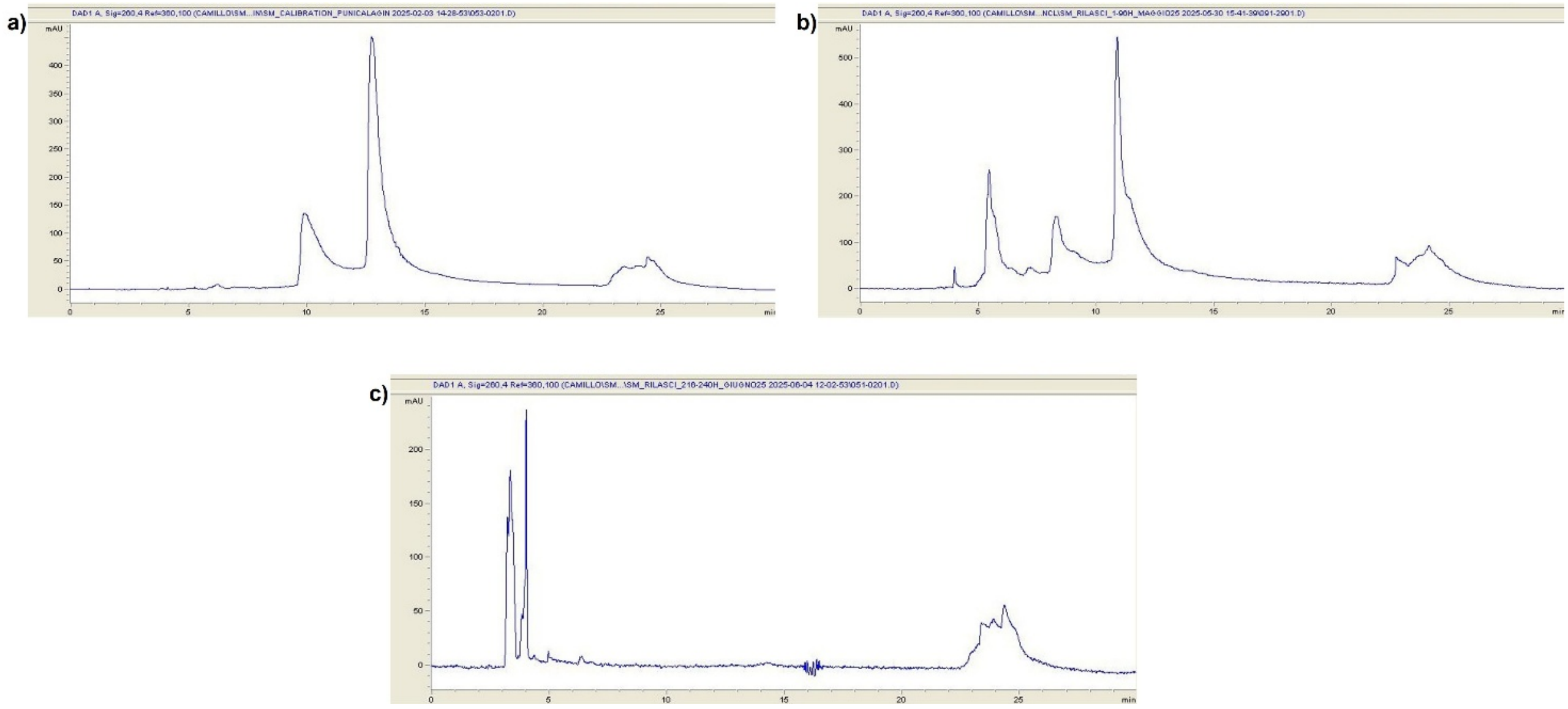

Figure S8. HPLC raw spectra of punicalagin: (a) punicalagin (α, β) calibration peaks; (b) punicalagin peaks during drug release analysis at 48 h, together with low-MW products from polymeric backbone due to S-S cleavage in GSH; (c) end of drug release analysis presenting only low-MW products from S-S cleavage in the polymeric backbone.

References:

[1] T. T. Wang, Z. H. Luo, Y. N. Zhou, *Macromolecules* **2023**, *56*, 1130.

[2] H. Pasch, M. I. Malik, in *Advanced Separation Techniques for Polyolefins*, Springer, Cham **2014**, Ch. 3.

[3] J. B. Matson, A. Q. Steele, J. D. Mase, M. D. Schulz, *Polym. Chem.* **2023**, *15*, 127.
